# Efficacy of Osteopathic Manipulative Treatment for Postoperative Recovery Following Total Knee Arthroplasty: A Meta-Analysis of Randomized Controlled Trials

**DOI:** 10.7759/cureus.100243

**Published:** 2025-12-28

**Authors:** Daniel P Oar, Jason S DeFrancisis, David Boesler

**Affiliations:** 1 Orthopaedic Surgery, Lake Erie College of Osteopathic Medicine, Bradenton, USA; 2 Osteopathic Medicine, Lake Erie College of Osteopathic Medicine, Bradenton, USA

**Keywords:** orthopaedic surgery, osteopathic manipulative medicine, osteopathic manipulative treatment (omt), total joint arthroplasty, total knee arthroplasty (tka), total knee replacement (tkr)

## Abstract

Total knee arthroplasty (TKA) is widely regarded as one of the most common and successful orthopaedic procedures in the United States, specifically in regard to the treatment of end-stage osteoarthritis of the knee. However, pain and range of motion (ROM) restrictions continue to burden patients in the acute postoperative period following TKA. Osteopathic manipulative treatment (OMT) is a safe and non-invasive therapy utilized by osteopathic physicians to treat and heal dysfunctions of the musculoskeletal system. The effectiveness of OMT in enhancing postoperative recovery remains a subject of ongoing debate. This meta-analysis investigates the efficacy of OMT in creating a difference in pain, measured on the numeric rating scale, and ROM in flexion in the acute postoperative period following TKA. Three randomized controlled trials (RCTs) with a total of 153 patients, 74 of them receiving OMT and 79 not receiving OMT, were identified and included in this meta-analysis. In regard to postoperative pain, the mean difference between OMT and non-OMT groups, using the random-effects model, was -0.59 (-1.96; 0.78). This mean difference favors a slightly lower postoperative pain level in the OMT group; however, this fails to reach statistical significance. Moderate to high heterogeneity was appreciated between studies (I²=50.8%), but this failed to reach statistical significance (p=0.1309). In regard to postoperative ROM in flexion, the mean difference between OMT and non-OMT groups, using the random-effects model, was 5.57 (-15.57; 26.70). This mean difference favors a slightly greater postoperative ROM in the OMT group; however, this fails to reach statistical significance. High heterogeneity was appreciated between studies (I²=89.9%), which proved to be statistically significant (p<0.0001). Given the overall similarity in patient recovery metrics, the effectiveness of OMT in improving postoperative pain and ROM following TKA cannot be proven at this time. However, the potential for clinical significance and minor improvements should be considered. Additional high-quality RCTs and comprehensive meta-analyses are needed to further define the role of OMT in the postoperative period following orthopaedic procedures.

## Introduction and background

Total knee arthroplasty (TKA) is one of the most widely performed orthopaedic procedures in the United States [1]. The incidence of TKA has significantly risen within the past 20 years, both nationally and globally, and with the ongoing rise in knee arthritis, it is projected that TKA will become even more common in the years to come [1,2]. TKA is often used to alleviate pain and enhance function in patients with end-stage, symptomatic knee osteoarthritis who have failed to respond to conservative measures such as physical therapy, pharmacological therapy, and intra-articular injections [3]. Primary knee osteoarthritis, which impacts nearly a quarter of adults over 40 years old, is the primary indication for TKA [4]. Osteoarthritis is characterized by progressive degeneration and loss of articular cartilage and subchondral bone, leading to chronic pain, joint deformity, and a loss of function [5]. TKA consists of resecting the diseased articular surfaces of the femur, tibia, and patella, followed by the implantation of metal and polyethylene prosthetic components that are designed to restore native knee function and relieve pain [6]. Patient satisfaction following TKA has increased to nearly 90%, climbing from earlier reports indicating satisfaction rates near 80% [7,8]. Despite satisfaction improvements, patients still have complaints after TKA, specifically in the early postoperative period [8]. Two of the most common complaints are residual pain and a limited range of motion (ROM), which can lead to unsatisfactory outcomes [8]. Initial postoperative issues, such as residual pain, limited ROM, swelling, and stiffness, can prolong recovery and even prolong hospital stays [9]. Due to the prevalence of TKA and the burden of early postoperative issues, the authors are interested in evaluating whether osteopathic manipulative treatment (OMT) could play a role in improving patient satisfaction outcomes even further.

OMT is a therapeutic approach employed by osteopathic physicians that utilizes various hands-on techniques to improve the physiologic function of the body [10]. OMT uses manual techniques to diagnose and treat somatic dysfunctions, which are areas of impaired or altered biomechanical function [10]. Goals of OMT include restoring native motion, alleviating pain, and optimizing overall health [10]. There is a wide range of OMT techniques, spanning from soft tissue manipulation to lymphatic pumping [10]. Clinically, one of the most common indications for OMT is musculoskeletal dysfunction and pain, including the management of chronic knee arthritis [10,11]. OMT has also been implemented in postoperative care, with particular relevance for patients recovering from orthopaedic procedures [12]. OMT can be utilized to improve edema, address pain, and reduce opioid use in postoperative patients following an orthopaedic surgical procedure [12,13]. A study conducted by Barral et al. focused on postoperative TKA patients and found that patients who received OMT had significantly less pain at rest and significantly less opioid consumption compared to patients treated with traditional postoperative measures that did not include OMT [13]. Given the potential benefits of OMT in chronic knee arthritis and its application in postoperative patients, further evaluation of OMT as adjunctive therapy in patients recovering from TKA is warranted [11].

To date, there has been limited data on the efficacy and specific role of OMT in influencing postoperative TKA outcomes. The purpose of this study is to evaluate the impact of OMT on postoperative pain, measured on the numeric rating scale (NRS), and ROM in flexion in patients who underwent TKA. By assessing if adjunctive treatment with OMT in the postoperative period following TKA leads to improvements in pain or ROM, this meta-analysis aims to determine the clinical value of integrating OMT into standard postoperative TKA protocols. If meaningful benefits are observed, OMT may prove to be a useful option in enhancing recovery and functional outcomes in post-TKA patients.

## Review

Materials and methods

Reporting 

The Preferred Reporting Items for Systematic Reviews and Meta-Analyses (PRISMA) criteria were followed for this review [14].

Research Question

Are there significant differences in postoperative pain or ROM when OMT is used in the acute postoperative period following TKA compared to standard rehabilitation protocols?

Inclusion Criteria

Studies meeting all inclusion criteria were eligible for this meta-analysis. Included studies compared an OMT intervention group directly to a non-OMT control group in the acute postoperative period following TKA. Only randomized controlled trials (RCTs) were eligible for inclusion. Studies must have collected data on postoperative pain using an NRS or ROM in flexion around postoperative day 4. Additionally, all studies had to be published in the English language. 

Exclusion Criteria

Excluded study designs included systematic reviews, retrospective studies, cross-sectional studies, qualitative studies, case reports, case series, and non-RCTs. All studies published in non-English language were excluded due to limitations preventing accurate translation. Additionally, all studies published prior to 2010 were excluded to ensure the currency of the included data. Studies with inadequate follow-up or failing to report either pain on NRS or ROM in flexion were excluded. All studies not related to OMT and TKA were not considered.

Search Strategy

A comprehensive literature search was conducted through October 2025 using the following databases: PubMed, Cochrane, Scopus, Google Scholar, ResearchGate, and American Osteopathic Association Osteopathic Research. Search terms consisted of "OMT", "osteopathic manipulative treatment", "osteopathic treatment", "TKA", "total knee arthroplasty", "postoperative pain", "pain reduction", "RCT", and "randomized controlled trial". This literature search resulted in 336 results, which were subsequently screened for inclusion and exclusion criteria. 

Data Sources

Manual searches of pertinent systematic reviews and meta-analyses were also performed to identify studies not captured in our initial search in October 2025.

Study Selection

A total of 336 studies were screened for eligibility based on predefined inclusion and exclusion criteria as outlined in previous sections. Two hundred and ninety-six articles were screened out for being published prior to 2010, unrelated to OMT following TKA, or being non-RCTs. Thirty-two records were also identified as duplicates and subsequently removed. This resulted in eight full-text articles available for further assessment. Five of these full-text articles were additionally excluded for not reporting pain on the NRS. Three full-text articles were therefore included in our final data analysis (n=3). No disagreements were found between reviewers (D.O. and J.D.).

Data Extraction

Data pertaining to title, author, year, sample size, study design, mean pain on NRS, mean ROM in flexion, standard deviation, and follow-up were extracted from each study. 

Quality Assessment 

To minimize potential bias, only RCTs were included in this study. Bias assessment was conducted utilizing the National Institute of Health (NIH) Study Quality Assessment Tool as well as with the use of funnel plots [15].

Statistical Analysis

Data analysis and graph production were performed using RStudio (v4.5.1, R Core Team, 2025) and the meta package (v7.1-0). Forest plots were generated for both pain on NRS and ROM in flexion to present each study's mean value, standard deviation, and associated confidence interval. A random-effects model, with 95% confidence intervals, was employed. Heterogeneity was assessed using the I² and Tau² statistics to evaluate variation across studies.

Assessment of Results

The forest plots included in this meta-analysis include calculations for both the random-effects and common-effects models. Subsequent sections of this manuscript will exclusively discuss the random-effects model, as it represents the most appropriate framework for analyzing the results of this study [16,17].

Results

Literature Search

**Figure 1:**
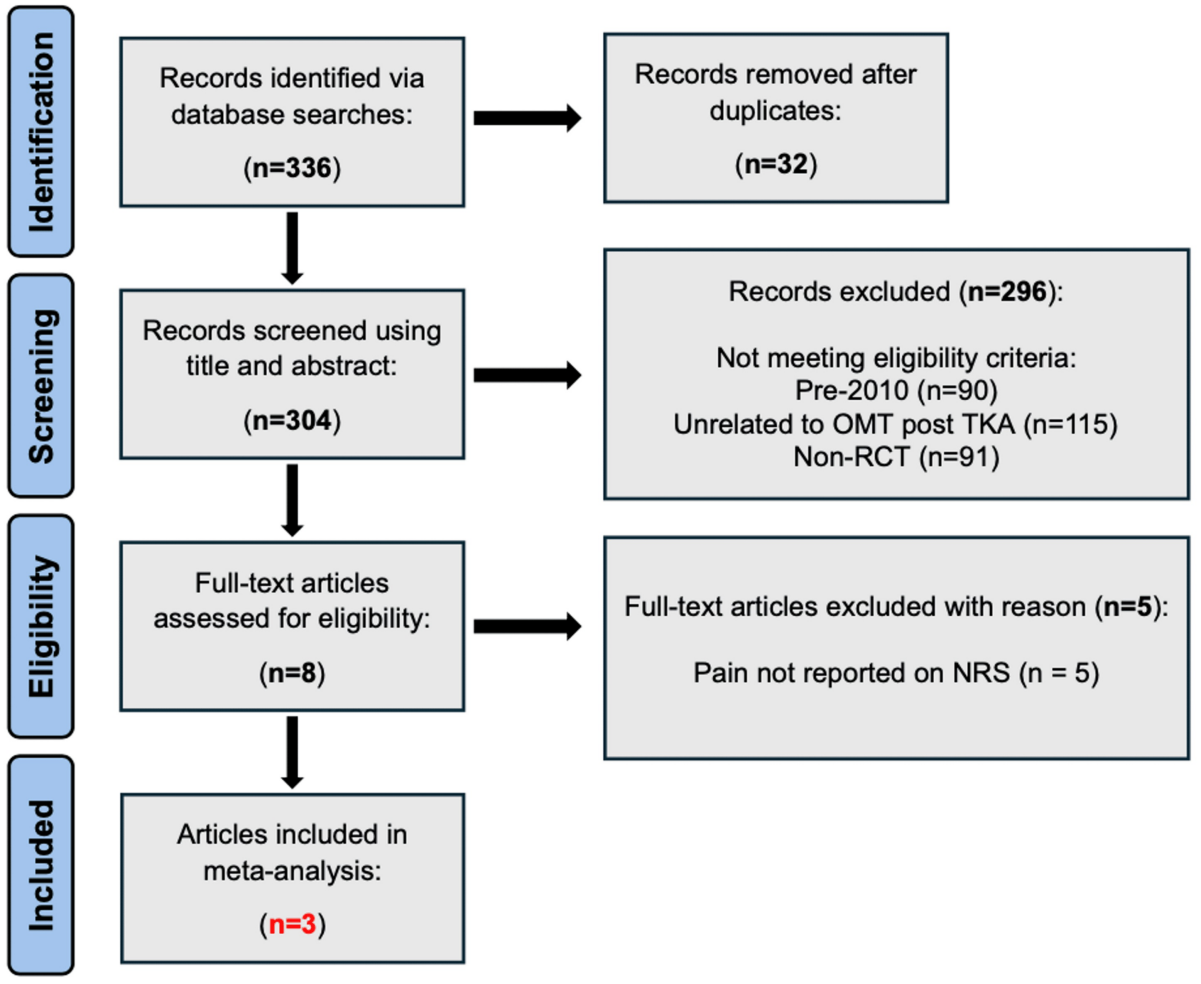
PRISMA flowchart displaying the literature search and article selection process n: number; OMT: osteopathic manipulative treatment; TKA: total knee arthroplasty; non-RCT: non-randomized controlled trial; NRS: numeric rating scale; PRISMA: Preferred Reporting Items for Systematic Reviews and Meta-Analyses

Upon our initial literature search, 336 records were identified. After extensive screening to identify only studies that met all eligibility criteria, three studies were identified and included in our data analysis (Figure 1) [18-20].

Characteristics of the Studies Included

The characteristics of all three studies included in this meta-analysis are summarized in Table 1 [18-20].

**Table 1:** Summary of the literature search and study characteristics included in the data analysis n: number; OMT: osteopathic manipulative treatment; RCT: randomized controlled trial; NRS: numeric rating scale; ROM: range of motion

Study	Total sample size (n)	OMT group size (n)	Non-OMT group size (n)	Study design	OMT technique utilized	Outcomes measured
Ebert et al., 2013 [18]	50	24	26	RCT	Manual lymphatic drainage	Pain on NRS, ROM in flexion
Tornatore et al., 2020 [19]	66	33	33	RCT	Manual lymphatic drainage	Pain on NRS, ROM in flexion
Karaborklu Argut et al., 2021 [20]	37	17	20	RCT	Joint and soft tissue mobilization	Pain on NRS, ROM in flexion

Risk-of-Bias Assessment

Risk-of-bias assessment was conducted using the NIH Study Quality Assessment Tool [15]. The quality assessment was conducted independently by two separate reviewers (D.O. and J.D.). No disagreements or conflicting results occurred between reviewers. Scoring was conducted by awarding 1 point for "yes" responses and 0 points for "no" responses to the NIH Study Quality Assessment Tool questions. An article was deemed "poor" if it was awarded 0-4 points, "fair" if it was awarded 5-9 points, and "good" if it was awarded 10-14 points. Results from this risk-of-bias assessment can be found in Table 2. The bias ruling for Ebert et al. was "good", the bias ruling for Tornatore et al. was "good", and the bias ruling for Karaborklu Argut et al. was "good" [18-20]. Funnel plots were also used to highlight the risk of publication bias for each outcome.

**Table 2:** Risk-of-bias quality assessment using the NIH Study Quality Assessment Tool Items 1-14 represent the 14 criteria questions used in the NIH Quality Assessment of Controlled Intervention Studies, a bias assessment tool which measures the methodological quality of each study. A "yes" or "no" answer is provided for each question [15]. N: no; Y: yes; NIH: National Institute of Health

Study	Bias ruling	Total score (out of 14)	1	2	3	4	5	6	7	8	9	10	11	12	13	14
Ebert et al., 2013 [18]	Good	12	Y	Y	Y	N	Y	Y	Y	Y	Y	Y	Y	N	Y	Y
Tornatore et al., 2020 [19]	Good	11	Y	Y	Y	N	N	Y	Y	Y	Y	N	Y	Y	Y	Y
Karaborklu Argut et al., 2021 [20]	Good	10	Y	Y	Y	N	N	Y	Y	N	Y	Y	Y	Y	Y	N

Findings 

Three RCTs were included in this meta-analysis (Figure 1). Among these three trials, a total of 153 patients in the postoperative period following TKA were assessed. Of these 153 total patients, 74 of them received OMT in the acute postoperative period, and 79 of them did not receive any OMT. The primary outcomes investigated in this meta-analysis include pain on the NRS pain scale and ROM in flexion, around postoperative day 4. Both outcomes were evaluated using the random-effects model.

**Figure 2:**
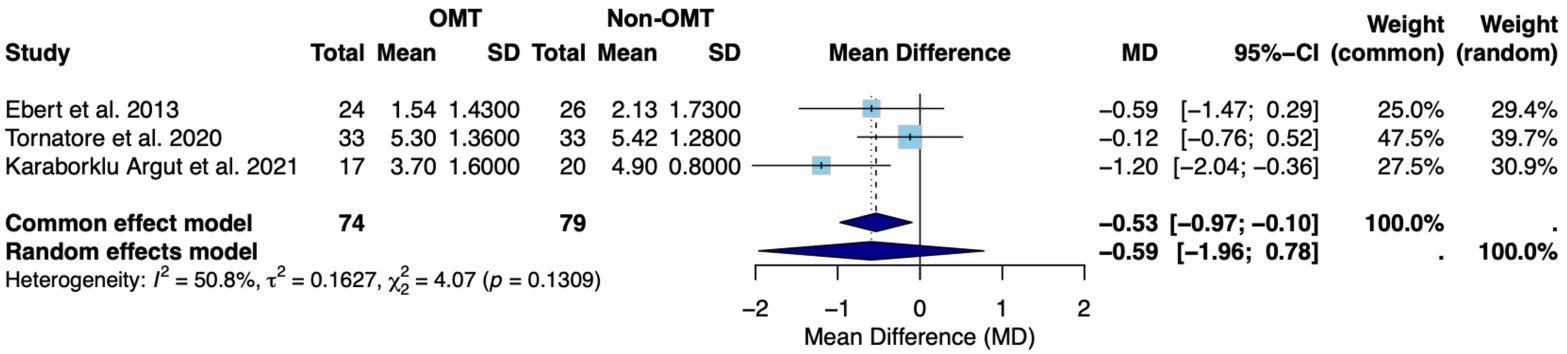
Forest plot of the mean difference in postoperative pain between OMT and non-OMT groups following TKA SD: standard deviation; CI: confidence interval; MD: mean difference; OMT: osteopathic manipulative treatment; TKA: total knee arthroplasty Studies included: Ebert et al., 2013 [18], Tornatore et al., 2020 [19], and Karaborklu Argut et al., 2021 [20]

Figure 2 presents the results of the random-effects model evaluating pain on the NRS pain scale on postoperative day 4 following TKA.

Figure 2 displays the random-effects model for pain assessed using the NRS pain scale on postoperative day 4 following TKA for Ebert et al. [18], Tornatore et al. [19], and Karaborklu Argut et al. [20]. The observed mean difference between the OMT and non-OMT groups was -0.59 (-1.96; 0.78) (Figure 2). Since this confidence interval includes zero, we can conclude that the random-effects model suggests that there was no statistically significant difference in pain on postoperative day 4 between the OMT and non-OMT groups (Figure 2). The overall effect favors a slightly lower postoperative pain level in the OMT group; however, this difference fails to reach statistical significance. The overall effect sizes varied between studies, with Tornatore et al. [19] contributing the highest weight in the random-effects model at 39.7% (Figure 2). Tornatore et al. [19] also had the narrowest confidence interval (-0.76; 0.52), indicating the greatest precision among analyzed studies (Figure 2). Heterogeneity was found to be moderate to high (I²=50.8%), but was not statistically significant (p=0.1309), indicating the null hypothesis of no heterogeneity could not be rejected (Figure 2) [21]. 

**Figure 3:**
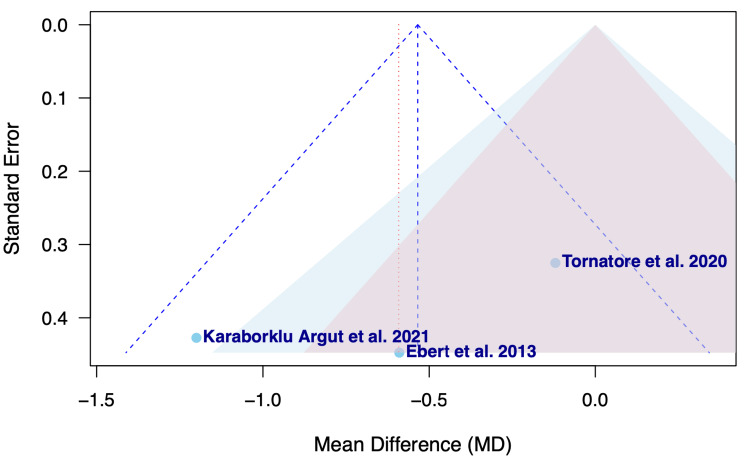
Funnel plot of publication bias in regard to postoperative pain between OMT and non-OMT groups following TKA OMT: osteopathic manipulative treatment; TKA: total knee arthroplasty Studies included: Ebert et al., 2013 [18], Tornatore et al., 2020 [19], and Karaborklu Argut et al., 2021 [20]

Figure 3 presents the funnel plot assessing publication bias among studies evaluating pain on the NRS pain scale on postoperative day 4 following TKA.

The funnel plot displayed in Figure 3 assesses the risk of publication bias among the studies in regard to postoperative pain [18-20]. A generally symmetrical distribution can be appreciated with effects pooled around the mean difference. Ebert et al. [18] and Karaborklu Argut et al. [20] appear near the lower portion of the plot with larger standard errors, reflecting their smaller sample sizes, with Ebert et al. [18] clustering close to the vertical midline. Tornatore et al. lies to the right of the pooled mean with a lower standard error, indicating a more precise estimate given the study's slightly larger sample size [19]. Overall, no study falls outside of the triangular funnel limits, and the positioning of the points suggests a low likelihood of publication bias for the outcome of postoperative pain (Figure 3).

**Figure 4:**
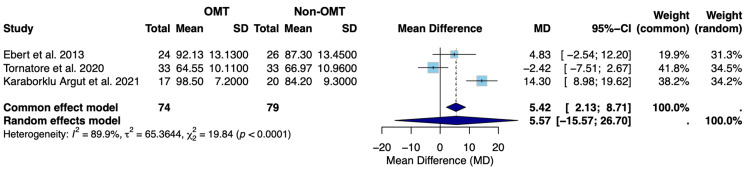
Forest plot of the mean difference in postoperative ROM in flexion between OMT and non-OMT groups following TKA SD: standard deviation; CI: confidence interval; MD: mean difference; ROM: range of motion; OMT: osteopathic manipulative treatment; TKA: total knee arthroplasty Studies included: Ebert et al., 2013 [18], Tornatore et al., 2020 [19], and Karaborklu Argut et al., 2021 [20]

Figure 4 presents the results of the random-effects model evaluating ROM in flexion on postoperative day 4 following TKA.

Figure 4 displays the random-effects model for ROM in flexion on postoperative day 4 following TKA for Ebert et al. [18], Tornatore et al. [19], and Karaborklu Argut et al. [20]. The observed mean difference between the OMT and non-OMT groups was 5.57 (-15.57; 26.70) (Figure 4). Since this confidence interval includes zero, we can conclude that the random-effects model suggests that there was no statistically significant difference in ROM in flexion on postoperative day 4 between the OMT and non-OMT groups (Figure 4). The overall effect favors a slightly greater ROM in flexion in the OMT group; however, this difference fails to reach statistical significance. The overall effect sizes varied only slightly between studies, with Tornatore et al. [19] contributing the greatest weight in the random-effects model at 34.5% (Figure 4). Tornatore et al. [19] also had the narrowest confidence interval (-7.51; 2.67), indicating the highest precision among analyzed studies (Figure 4). Heterogeneity was found to be high (I²=89.9%), which proved to be statistically significant (p<0.0001), indicating rejection of the null hypothesis of no heterogeneity (Figure 4) [21].

**Figure 5:**
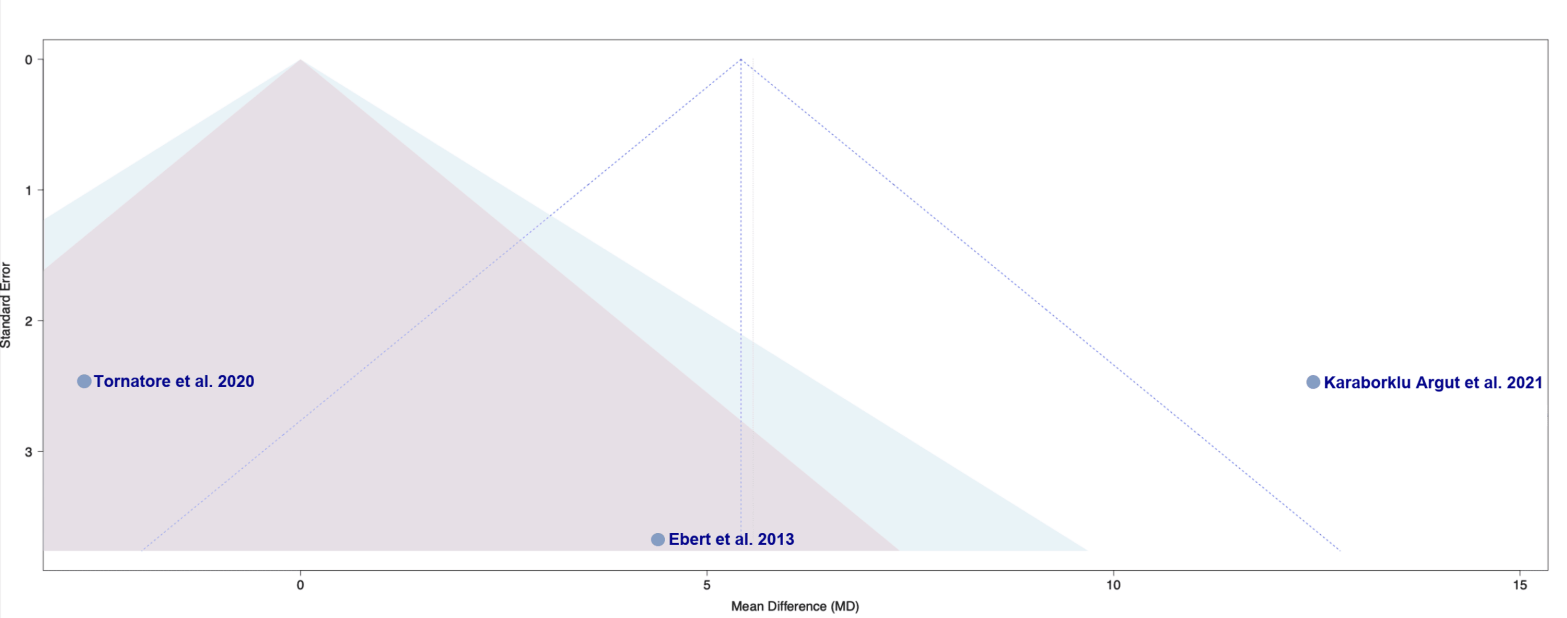
Funnel plot of publication bias in regard to ROM in flexion between OMT and non-OMT groups following TKA ROM: range of motion; OMT: osteopathic manipulative treatment; TKA: total knee arthroplasty Studies included: Ebert et al., 2013 [18], Tornatore et al., 2020 [19], and Karaborklu Argut et al., 2021 [20]

Figure 5 presents the funnel plot assessing publication bias among studies evaluating ROM in flexion on postoperative day 4 following TKA.

The funnel plot displayed in Figure 5 assesses the risk of publication bias among the studies in regard to postoperative ROM in flexion [18-20]. This funnel plot displays some notable asymmetry between the analyzed studies. Tornatore et al. sits on the left side of the plot and has a relatively low to moderate standard error, indicating a slightly larger sample size with more precise results [19]. Ebert et al. lies near the bottom center of the funnel with the largest standard error, suggesting it is the least precise study in the analysis. However, its effect remains close to the vertical midline [18]. In contrast, Karaborklu Argut et al. is positioned relatively far to the right of the triangular funnel limits, with a large positive mean difference and low to moderate standard error, representing a study with a notably larger reported effect [20]. Given that these studies are relatively unevenly distributed and one point lies outside the triangular funnel limits, the plot suggests that for the outcome of postoperative ROM in flexion, results should be interpreted with caution (Figure 5).

Discussion

This meta-analysis investigated whether OMT caused a significant difference in pain levels or ROM in flexion in the acute postoperative period following TKA. OMT techniques varied between studies and consisted of manual lymphatic drainage as well as joint and soft tissue mobilization (Table 1). A total of three RCTs, consisting of 153 total patients, were assessed in this study, with a total of 74 patients receiving OMT and 79 patients not receiving OMT (Table 1).

Both OMT and non-OMT groups had similar pain levels and degrees of ROM in flexion on postoperative day 4, indicating that OMT did not make a statistically significant difference in reducing postoperative pain levels or improving ROM (Figure 2 and Figure 4). Overall, across all three studies, there was an average NRS pain score of 0.59 (-1.96; 0.78) points lower in the OMT group compared to the non-OMT group using the random-effects model (Figure 2). However, the association failed to achieve statistical significance; the confidence interval encompassed zero, suggesting insufficient evidence to conclude a meaningful difference between groups. A moderate to high degree of heterogeneity was also recognized (I^2^=50.8%). However, this failed to reach statistical significance (p=0.1309) (Figure 2). In regard to postoperative ROM in flexion, pooling all three studies under the random-effects model revealed an average increase of 5.57 (-15.57; 26.70) degrees in the OMT group relative to the non-OMT group (Figure 4). However, this association failed to reach statistical significance, given that the confidence interval encompassed zero, suggesting there was insufficient evidence to conclude that this increase was a meaningful difference. A high degree of heterogeneity was observed for this outcome measure (I²=89.9%), which was statistically significant (p<0.0001), reflecting significant variability among the included studies (Figure 4). The observed heterogeneity is likely multifactorial in origin, potentially stemming from variation in the specific OMT techniques employed, differences in physician proficiency and technique execution, as well as surgical factors such as surgeon experience and implant selection. However, studies have concluded that meta-analyses with a limited number of included studies and smaller sample sizes may possess larger variations in I^2^ value, regardless of true heterogeneity [21]. Results should therefore be interpreted with caution.

Although no statistically significant differences in pain or ROM were found, it is important to consider the possibility of clinical significance. Clinical significance remains an important consideration when evaluating the effect of OMT on pain and ROM in the acute postoperative period following TKA. Small improvements in pain or early joint mobility may not reach statistical thresholds in studies with limited sample sizes, yet these changes can still be meaningful for patient recovery. Even modest reductions in pain may decrease opioid requirements, facilitate earlier participation in rehabilitation, and improve functional milestones such as ambulation and transfers [22]. Similarly, slight gains in ROM during the early postoperative period may accelerate recovery trajectories and reduce stiffness or long-term mobility restrictions [23]. Therefore, the absence of statistical significance does not necessarily negate the potential for clinical value, particularly in a postoperative population where incremental benefits can meaningfully influence recovery and outcomes. Continued research with more high-quality RCTs is needed to further investigate the utility of OMT in the postoperative period and draw stronger conclusions about its effectiveness in decreasing pain and improving ROM following TKA.

The results of this meta-analysis add to the growing but still relatively limited number of studies on the use of OMT in the postoperative period. OMT has demonstrated potential postoperative benefits, including reduced rates of ileus, shorter hospital stays, and lower pain levels [12]. However, these findings have not been consistently reproduced across studies, and the overall effectiveness of OMT in this setting remains a topic of ongoing debate [12]. A study by Kim et al. investigated the use of OMT in patients following lumbar microdiscectomy and found that patients receiving postoperative OMT saw a residual leg pain decrease of 53%, while patients receiving standard exercise therapy only experienced a 17% reduction in postoperative leg pain [24]. Similarly, a retrospective study by Crow and Gorodinsky found that OMT was associated with a significantly shorter length of hospital stay in patients following abdominal surgery [25]. Although OMT has demonstrated promising postoperative effects, the evidence is variable, and additional rigorous, high-quality studies are required to determine its definitive role in postoperative care.

Overall, the results of this study did not show any statistically significant benefits of OMT in the acute postoperative period following TKA; however, clinical significance should be considered. Although this meta-analysis yields significant insights, several notable limitations should be discussed, and results should be interpreted with caution, given the relatively small sample size, limited number of included studies, and notable heterogeneity found for both outcome measures. It should also be noted that although outcome measurements were centered around postoperative day 4, Karaborklu Argut et al. measured pain and ROM on patient discharge, which was on average postoperative day 4.57±1.32 and 4.62±0.97 in the OMT and non-OMT groups, respectively. This may have led to small variations in reported outcomes. Additionally, standardization between studies remains a concern, especially for ROM in flexion, which had a statistically significant degree of heterogeneity (p<0.0001) (Figure 4). Given that interventions were not identical and the specific OMT techniques varied among studies, methodological variability may have contributed to increased heterogeneity in the observed outcomes.

Future studies should ensure standardized follow-up periods, consistent OMT protocols, and larger sample sizes to improve methodological rigor and statistical power to draw more definitive conclusions on the use of OMT in the acute postoperative period following TKA. Subsequent studies should also assess the effects of OMT on a wider variety of outcome measures and patient satisfaction metrics to gain a more complete understanding of its utility in the postoperative period. Continued research efforts should also investigate the efficacy of OMT postoperatively following other common orthopaedic procedures such as total and hemiarthroplasty of the hip, anatomic and reverse total shoulder arthroplasty, and fracture fixations. As additional high-quality RCTs are conducted, subsequent meta-analyses will be better equipped to clarify and strengthen our understanding of the efficacy of OMT in the postoperative setting.

## Conclusions

This meta-analysis found no statistically significant differences in postoperative pain on the NRS pain scale or ROM in flexion between OMT and non-OMT groups following TKA. Both OMT and standard postoperative interventions resulted in comparable pain levels and ROM in flexion, suggesting relatively equivalent effectiveness in improving these postoperative metrics. Given the overall similarity in results, this study indicates that OMT is not yet supported as a proven method to reduce pain and ROM restrictions in the acute postoperative period following TKA. However, clinical significance should be considered as minor improvements in pain and ROM may still be beneficial to patients in the postoperative period, even if these improvements fail to reach statistical significance. The choice to implement OMT in a patient's postoperative plan following TKA should be guided by physician and patient preference, comfort level, and experience. Additional high-quality RCTs are needed to establish more definitive conclusions regarding the efficacy of OMT in the postoperative period following TKA, as well as other common orthopaedic procedures. Future studies should incorporate larger sample sizes, a broader range of OMT techniques, standardized treatment protocols, and consistent follow-up. As the role of OMT in postoperative management becomes more defined, clinicians will be better equipped to provide more effective, evidence-based postoperative care.
